# Drug Screening, Oral Bioavailability and Regulatory Aspects: A Need for Human Organoids

**DOI:** 10.3390/pharmaceutics13081280

**Published:** 2021-08-17

**Authors:** Tamara Zietek, Wolfgang A. D. Boomgaarden, Eva Rath

**Affiliations:** 1 Doctors against Animal Experiments, 51143 Köln, Germany; 2 PharmaInformatic Boomgaarden, 26723 Emden, Germany; wolf@pharmainformatic.com; 3 Chair of Nutrition and Immunology, Technische Universität München, 85354 Freising, Germany

**Keywords:** intestinal organoids, regulatory aspects, nutrient absorption, drug uptake, non-animal models, 3R, intestinal transport processes, intestinal epithelial cells

## Abstract

The intestinal epithelium critically contributes to oral bioavailability of drugs by constituting an important site for drug absorption and metabolism. In particular, intestinal epithelial cells (IEC) actively serve as gatekeepers of drug and nutrient availability. IECs’ transport processes and metabolism are interrelated to the whole-body metabolic state and represent potential points of origin as well as therapeutic targets for a variety of diseases. Human intestinal organoids represent a superior model of the intestinal epithelium, overcoming limitations of currently used in vitro models. Caco-2 cells or rodent explant models face drawbacks such as their cancer and non-human origin, respectively, but are commonly used to study intestinal nutrient absorption, enterocyte metabolism and oral drug bioavailability, despite poorly correlative data. In contrast, intestinal organoids allow investigating distinct aspects of bioavailability including spatial resolution of transport, inter-individual differences and high-throughput screenings. As several countries have already developed strategic roadmaps to phase out animal experiments for regulatory purposes, intestinal organoid culture and organ-on-a-chip technology in combination with in silico approaches are roads to go in the preclinical and regulatory setup and will aid implementing the 3Rs (reduction, refinement and replacement) principle in basic science.

## 1. Introduction

Oral bioavailability refers to the extent a substance or drug becomes completely available to systemic circulation or to its intended biological destination(s) via the oral route [1]. High oral bioavailability reduces the amount of a drug necessary to achieve a desired pharmacological effect, therefore reducing the risk of side-effects and toxicity. Low oral bioavailability results in low efficacy and is a major reason for drug candidates failing to reach the market. Hence, oral bioavailability is one of the most important properties in drug design and development.

Critical determinants of oral bioavailability include the gastrointestinal (GI) tract physiology, hepatic first-pass metabolism, plasma protein binding and excretion via the kidneys. In the GI tract, the intestinal epithelium crucially contributes to oral bioavailability by constituting an important site for drug absorption, distribution, metabolism, and excretion (ADME). Exposed to high concentrations of food-borne, microbiota-derived, or other exogenous xenobiotics, including toxins and carcinogens, intestinal epithelial cells (IEC) are trained to defend themselves and the body from harmful substances. Hence, expression of enzymes and transporters involved in drug metabolism and xenobiotic defense including cytochrome P450 isoenzymes (CYPs), UDP-glucuronosyltransferase isoforms (UGTs) and ATP binding cassette (ABC) transporters, e.g., the multidrug efflux pump P-glycoprotein (P-gp), give rise to the concept of the intestinal epithelium as a pharmacogenetic barrier [2]. Additionally, the inert function of enterocytes, the subtype of IECs specialized in uptake processes, to absorb nutrients, contributes to oral bioavailability of drugs. For example, certain inhibitors of angiotensin-converting enzyme, protease inhibitors, antivirals, and peptidomimetics like β-lactam antibiotics are absorbed by the peptide transporter 1 (PEPT1) [3]. The main route of orally administered drugs into the systemic circulation is by direct passage from the IEC layer into the mesenteric blood capillaries, yet certain drugs gain access to the systemic circulation via intestinal lymphatic absorption. Following this pathway, absorbed drugs associate with fats and lipoproteins within IECs as they are processed into chylomicrons. [4]. Vice versa, enterocyte fatty acid oxidation (FAO), which has been implicated in the in the control of eating, can be pharmacologically modified [5,6]. Consequently, intestinal transport processes, IEC metabolism and their interconnection to whole-body metabolic state are relevant to a variety of diseases and represent potential therapeutic targets. Next to intestinal pathologies like malabsorption syndromes or inflammatory diseases, these pathologies comprise metabolic disorders such as obesity and type 2 diabetes, and additionally, diseases treated with drugs and prodrugs that are actively absorbed and/or metabolized by enterocytes. Thus, beyond constituting a physical barrier separating the host from its environment including the intestinal microbiota, IECs actively serve as gatekeepers of nutrient availability and metabolic health for the whole organism.

Despite this importance, many aspects of nutrient absorption, enterocyte metabolism, and drug bioavailability are still unknown, e.g., the underlying causes of fructose malabsorption remain elusive [7]. Hence, there is a need for advanced model systems that enable studying intestinal transport processes and IEC metabolism, especially in the context of drug development. Commonly used in vitro models of the intestinal epithelium, such as Caco-2 cells or rodent explant models (Ussing chamber, everted gut sac models) are of limited value due to their cancer and non-human origin, respectively. In particular, species differences result in poorly correlative data, and findings obtained in these models cannot be translated reliably to humans. Contrarily, human intestinal organoids allow investigating different aspects of oral bioavailability, form inter-individual differences to broad uptake screenings, thus representing a superior model of the intestinal epithelium. Intestinal organoid lines created from biopsies of healthy and diseased individuals [8] can be readily expanded and maintain their region-specific expression patterns upon differentiation. Functional characteristics including absorption studies and metabolomics have already been conducted on three-dimensional organoids derived from human small and large intestinal biopsies [9]. Cultured in 2D on trans-well membranes, organoid-derived cell lawns provide a valuable tool to study drug permeability in spatial resolution as well as inter-individual differences. Of note, intestinal organoids not only enable measurement of intestinal transport and intracellular (drug-) metabolism in a dynamic way, downstream events like molecular signalling pathways resulting in hormone secretion can be investigated in parallel [10].

Several countries have already developed strategic roadmaps to phase out animal experiments for regulatory purposes. This increases the need for a timely substitute that reflects physiology in the best possible manner. Intestinal organoid culture and organ-on-a-chip technology are promising ways forward in the preclinical and regulatory setup and will aid implementing the 3Rs (reduction, refinement and replacement) principle in basic science.

In this review, we briefly describe the role of IECs for oral bioavailability and discuss the (future) contribution of human intestinal organoid culture and organ-on-a-chip technology for basic research, drug screening and the regulatory setup, illustrating the advantages of a human-based model for drug development.

## 2. Bioavailability and the Intestinal Epithelium

### 2.1. Anatomy and Function of the Intestinal Epithelium

The intestinal epithelium is a single-cell layer located in close proximity to a complex milieu of dietary factors, digestive fluids and a dense microbial ecosystem. Simultaneously, this multicellular interface composed of IECs not only forms a barrier to protect the host from adverse components of this luminal environment, mounting physical and immune-mediated defence mechanisms, but also orchestrates nutrient digestion and uptake as well as metabolic functions [11]. Distinct, specialized IEC subtypes arise from intestinal stem cells that constantly renew the epithelial layer. Next to the most abundant IEC subtype, absorptive enterocytes, that take up nutrients and ions, this comprises cell of the secretory lineage. Paneth cells provide factors to maintain the stem cell niche at the bottom of the crypts and produce antimicrobial peptides; mucin-producing goblet cells create a physical barrier by generating a mucus layer covering the epithelium; enteroendocrine cells (EEC) secrete different hormones to control bowel movement and regulate metabolism; and taste-chemosensory tuft cells participate in immune responses [12,13]. The complete IEC layer, forming an area of 30–40 m^2^, is renewed every 3–5 days, and with approximately 1400 cells being shed from a single villus tip per day, the small intestinal epithelium shows the highest turnover rate of any fixed-cell population in the body. This enormous regenerative capacity of intestinal stem cells most likely represents a protective mechanism against injuries and infections [12,14], but beyond physiological function, it also allows efficient culture of intestinal organoids. Although the epithelium of the small and large intestine share similar basic anatomical architecture, their biological functions and tertiary architecture are noticeably different. The length of villi decreases from the proximal to distal small intestine with the colonic epithelium presenting a flat luminal surface with multiple crypts, reflecting the compartmentalized requirements regarding absorptive functions. Different subtypes of IECs are only found in distinct locations of the intestine. For example, different classes of EEC producing different patterns of hormones are distributed along the gastrointestinal tract, and anatomical sites of absorption differ for nutrients and drugs [15,16]. As intestinal stems cells are intrinsically programmed with their location-specific functions [17], organoids derived from different regions of the gastrointestinal tract retain the unique properties of their site of origin, enabling a new dimension of spatial resolution in research, not covered previously by cell lines. Also the pH varies greatly along the gastrointestinal tract, increasing from pH 1.2 in the empty stomach, pH 5–7 in the small intestine, to 6–7.5 in the colon [18]. Digestion starts in the oral cavity with salivary amylase and continuous in the stomach, however, the small intestine is the major site for both digestion and absorption [19]. Microvilli, small (1µm) protrusions covering the luminal side of enterocytes, increase the surface available for digestion and absorption about 600-fold, and along with the associated glycocalyx (a mixture of mucus, proteins and cholesterol anchored to the membrane) forms the so-called brush border. The brush border membrane constitutes the major site of nutrient absorption and an important physical and enzymatic barrier to drug absorption [20,21]. As mentioned before, most nutrient and mineral uptake takes place in the small intestine, hence, most nutrient transporters are predominantly expressed in the duodenum and jejunum [22,23]. Yet, before reaching the brush border, luminal presystemic drug metabolism can be an important factor for oral bioavailability of certain drugs.

### 2.2. Presystemic Luminal Drug Metabolism

**Figure 1:**
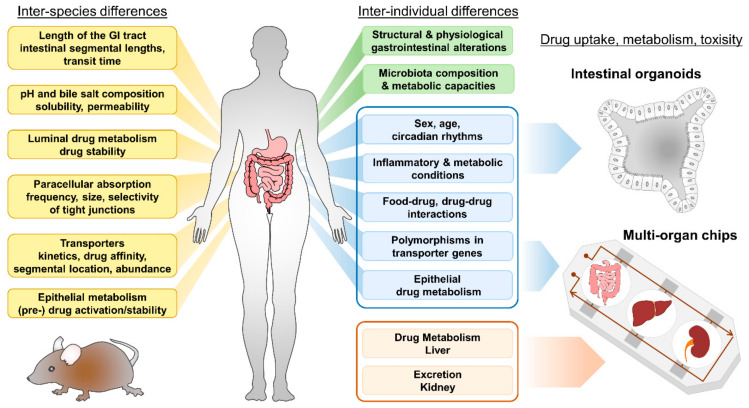
Inter-species and inter-individual differences accounting for differences in oral bioavailability of drugs. Human intestinal organoids and multi-organ chips (MOC) facilitate research including personalized-medicine approaches and drug screening. Aspects given in blue can be modelled by intestinal organoids, systemic (liver) metabolism and excretion (kidney) properties (depicted in light red) can be implemented using MOCs.

Luminal pH and the presence of digestive enzymes, as well as the segmental location of intestinal metabolism, profoundly affect drug dissolution and absorption [24], and differences in luminal presystemic drug metabolism contribute to observed interspecies inconsistencies in oral bioavailability [25] (Figure 1). Furthermore, food-induced changes in the bioavailability of certain drugs can cause clinically relevant food–drug interactions by altering the pharmacokinetics and/or pharmacodynamics of the respective medication. These food–drug interactions can occur on different levels of specificity: the physiological response to food intake, particularly gastric acid and bile acid secretion; altered absorption with fatty, high protein and fiber diets; chelation with components in food; competition for transport; and effects of food components on drug-metabolizing enzymes [26,27]. The latter have gained attention with the famous example of grapefruit juice decreasing CYP3A4 activity in small intestinal epithelial cells [28].

Additionally, the composition of the intestinal microbiota and its functional capacity (referred to as the microbiome) impacts drug bioavailability [29]. Even though most bacteria are found in the colon, the bacterial load in the small intestine is estimated to increase from 10^3^ colony forming units per ml in the duodenum to 10^7^ in the ileum [30], and the small intestinal microbiota profoundly contributes to nutrient bioavailability and vitamin synthesis [31]. Despite some important observations from xenobiotic metabolism in general, the extensive direct and indirect effects of the gut microbiome on drug metabolism and efficacy is currently underappreciated in clinical studies evaluating the pharmacokinetics of new drugs. To date, there is only an incomplete understanding of how, and to what extent, the microbiome influences drug metabolism and absorption, and this relates to systemic concentrations of parent compounds and toxic metabolites [30]. So far, attempts to incorporate microbiota into intestinal organoids mainly comprise selected commensals or pathogens, and co-culture with organoids creates a unique environment offering a possibility to grow species that have previously been difficult to grow and maintain [32,33]. In addition, microbiome-derived metabolites have been tested for their effects on intestinal organoids [33,34]. Presystemic luminal drug metabolism can be investigated using in vitro intestinal fermentation models designed to study compound digestion and production of metabolites [35]; hence, merging models could help better understanding the complex interaction of microbiome, drug metabolism, and host effects.

In humans, several factors account for inter- and intra-individual differences in oral bioavailability. These include, but are not restricted to: GI surgery or chronic inflammatory intestinal conditions [36], co-administration of drugs [37], food–drug interactions, feeding condition, age and sex [38] of the patient, ethnic background [39] and the patient’s unique physiology (including phenotypic polymorphisms) [40] (Figure 1). In particular, polymorphisms in transporter genes affecting expression or activity can influence bioavailability of drugs and toxins and may also predispose to certain diseases [2]. As human intestinal organoids conserve the phenotype of the donor [41], they are superior tools for investigating aspects accounting for individual capacities of drug uptake (Figure 1, depicted in blue).

## 3. Intestinal Nutrient and Drug Transport

Nutrient and drug absorption are interrelated and share common mechanisms. Crossing the intestinal epithelium to reach the systemic circulation can be achieved via paracellular or transcellular mechanisms, including active transporters [42]. Yet, in contrast to nutrients, expression of efflux pumps such as ABC transporters in IECs critically also contributes to drug bioavailability [2]. In the context of drug absorption, passive diffusion was long considered to be the dominant route of permeation. However, the coexistence of passive and transporter-mediated processes in drug uptake is more and more appreciated, and for particular drugs, absorption via transporters is supposed to be the main route of permeation [43]. Drug-intrinsic properties further determine the route of passive diffusion, with nonpolar molecules being absorbed transcellularly and polar compounds typically permeating paracellularly through the aqueous pores created within the tight junctions (TJs) [44]. Of note, the surface area for the transcellular pathway in the intestine is over 1000 times larger than that available for paracellular transport [45]. Efficient uptake via transporters requires a fine-tuned network of transport proteins, ion pumps and cellular metabolism. Due to the inherent changes of cell lines compared to the native cells (cancer- or embryonic origin, genetic modifications to increase survival and proliferation in vitro, but hampering full differentiation) these complex network are not reproduced appropriately. For instance, Caco-2 cells do not display physiological transporter expression, in contrast to intestinal organoids, that show expression patterns of transporters and ion pumps comparable to their site of origin [9].

### 3.1. Intestinal Nutrient Absorption

All three macronutrients, carbohydrates, amino acids, and fats, possess specific transporters for absorption. Lipid absorption involves emulsification and hydrolysis of dietary fat in the lumen of the intestine, along with micelle formation. Micelles facilitate the transfer of cholesterol, phospholipids, free fatty acids and other constituents of dietary fats as well as lipid-soluble vitamins across the unstirred water layer to the enterocyte membrane. For the uptake and transport of the diverse components of the micelles across the apical membrane of the enterocyte, protein-independent diffusion model and protein/transporter-dependent mechanisms have been proposed [46]. Within the enterocytes, lipids are resynthesized and assembled into chylomicrons and high density lipoproteins for secretion [47]. The physiological pathway of fat digestion and uptake has gained increasing attention in drug development, as micelles can act as nanocarriers promoting the oral absorption of hydrophobic drugs and nutrients [21,48,49], and association of drugs with chylomicrons might prevent metabolic inactivation by avoiding the hepatic first-pass liver metabolism, as chylomicrons are transported via the lymphatic circulation [4]. Alternatively, enterocyte lipid metabolism may be targeted pharmacologically to lower systemic lipid load and energy intake, thus affecting hyperlipidemia, obesity, metabolic syndrome, steatosis, insulin resistance, atherosclerosis and other disorders [5,47].

### 3.2. Intestinal Transporters and Drug Uptake

The function of cellular transporters can be categorized into facilitated diffusion and active transport. While primary active transporters typically use ATP to translocate molecules across membranes and against their concentration gradient, facilitated diffusion uses either protein channels or secondary active carrier proteins. Carrier proteins catalyze simultaneously the facilitated diffusion of a driving solute (usually an ion) and the active transport of a given solute. The electrochemical gradient of the driving solute provides the force for active transport of the target substrate, and depends on the electrochemical potential difference created by primary active ion pumps [50]. Many nutrient transporters act by facilitated diffusion. Hence, a complex network of different transporters is needed to ensure efficient uptake and efflux of nutrients (and drugs), and this interdependence contributes to the fact that many aspects of intestinal absorption are still not fully understood, owing to the before-mentioned drawbacks of commonly used cell lines. The superior properties of intestinal organoids will help gain a better understanding of these basic biological processes in the future.

In human physiology, solute carrier (SLC) transporters within the major facilitator superfamily (MFS) contribute to the transport of nutrients, metabolites, and other substrates and are crucial for growth, metabolism, and homeostasis. Yet, of the >500 recognized SLC transporters, up to 30% are considered orphans in that their function remains unknown, despite their importance to cellular physiology and drug development [51,52]. Additionally, identification of a particular substrate for transport in vitro does not exclude other physiological substrates to be transported by the same transporter. For example, 14 different isoforms of facilitative glucose transporters (GLUT) (GLUT1–GLUT14) have been identified in humans with partly overlapping substrate preferences and kinetics and the reason for this redundancy remains elusive [53]. Both, members of the GLUT family and members the sodium glucose co-transporter (SGLT) family are involved in sugar uptake, but the specificity of some of these transports is still unknown, contributing to the fact that intestinal sugar absorption is still not fully understood [54]. Mutations in SGLT1 (encoded by *SLC5A1*) and mutations in GLUT2 (encoded by *SLC2A2*) cause glucose–galactose malabsorption and Fanconi–Bickel syndrome, respectively, and furthermore, genetic variants in other transporters are also associated with human diseases [55,56]. In particular, fructose uptake mediated by GLUT5 has been a focus of attention, as increasing fructose consumption in the last decades is associated with developing cardiovascular diseases and type 2 diabetes [57]. Also in this case, even if defective absorption is most likely, the molecular basis of fructose malabsorption could not be characterized yet. The possibility to use patient-derived organoids might foster unraveling mechanisms contributing to defective fructose transport.

Intestinal peptide transport is of particular relevance to drug uptake. Peptide transport over the plasma membrane is coupled to proton cotransport enabling transport of di- and tripeptides against a substrate gradient [24]. The oligopeptide transporters PEPT1 and PEPT2 (encoded by *SLC15A1* and *SLC15A2* are highly expressed in the intestine and have been shown to aid the absorption of orally administrated drugs [58]. PEPT1 facilitates absorption of certain inhibitors of angiotensin-converting enzyme (ACE), protease inhibitors, and antivirals [43,59,60,61]. Drugs such as the antivirals valacyclovir and valganciclovir even have been modified into so-called “pro-drugs” to improve their adsorption by PEPT1 [58,62,63]. Of note, also peptidomimetics such as aminocephalosporins are substrates of PEPT1. Peptidomimetics are “compounds mimicking a peptide or protein, which possess the ability to interact with a biological target to exert agonistic or antagonistic effects” [64,65]. They have a great potential in drug discovery, exerting antimicrobial activities and other drug-like properties [42,66]. For instance, peptidomimetics have been designed for cancer therapy, e.g., to sensitize cancer cells to chemotherapeutics [67], induce apoptosis [68], and to specifically target integrins for interfering with angiogenesis and other aspects of tumour biology [69,70]. Primary goals in the development of orally available peptides are enhancing their stability to enzymatic degradation and improving their intestinal transport. Common strategies to improve bioavailability comprise cyclization of functional peptide motifs, the use of d- instead of l-amino acids and *N*-methylation [42]. Cilengitide, an anticancer drug acting as an inhibitor of α V integrins, is an example of such a modified cyclic pentapeptide. It possesses one d- amino acid and one *N*-methylation and consequently, Cilengitide is completely stable in humans and excreted with a half-life of 4 h without any metabolization [71]. However, intestinal permeation from the lumen into the bloodstream remains a major challenge for drug design. Even small structural changes to the backbone/scaffold containing the active peptide motive affect intestinal and cellular permeability. A change in one methyl position can greatly impact permeability properties, and hence modifications to improve affinity or selectivity might have negative effects on intestinal absorption [72]. As intestinal uptake routes and the transporters involved are not clarified in detail for such peptide drugs, it is not possible to reliably predict the impact of such chemical modifications on the transport/oral bioavailability of drug candidates [73]. Hence, drug development depends on screening systems that ideally closely reflect epithelial transport physiology like intestinal organoids.

### 3.3. Intestinal Ion Pumps

For continuous uptake via secondary active transporters, IECs need to maintain the transmembrane ionic gradients via ion pumps. Additionally, augmented or prolonged acidification of the cell through proton symport, e.g., by peptide transporters, has to be avoided, thus, surplus protons are directly exported in exchange with Na^+^ by sodium-proton exchangers (NHEs). Several types of NHEs are expressed in enterocytes and in particular, NHE3 is required for proper PEPT1-mediated transport [74]. While NHE3 is predominantly involved in epithelial transport processes in the small intestine, the epithelial sodium channel (ENaC) plays a profound role in the large intestine. Following the region-specific expression of primary nutrient transporters in organoids, these ion transporters also resemble the physiological expression patterns [9]. These transporters linked to nutrient absorption play crucial roles in physiological uptake processes and under disease (diarrhoea) conditions and are targeted by clinically relevant drugs as well as bacterial toxins [75]. The same is true for organic anion transporters (OATs) and organic cation transporters (OCTs), which belong to the SLC22 family and are expressed in the intestine (next to liver, brain, and kidney). They possess a broad substrate specificity, including differently charged organic ions as well as other molecules. Thus, the SLC22 family mediates transport of a diverse range of compounds, including anionic peptides, bile acids, steroid conjugates, thyroid hormones, as well as numerous drugs, e.g., statins, nonsteroidal anti-inflammatory (NSAID) drugs, and other xenobiotic substances [76]. Accordingly, several of these transporters of the MFS superfamily not only transport certain drugs, affecting the pharmacokinetics of orally administrated drugs, but are targeted themselves by FDA-approved drugs [77]. Furthermore, their importance for the pharmaceutical industry is highlighted by the fact that the FDA recommends several of these transporters to be tested for the transport of new drugs [78].

## 4. Intestinal Epithelial Cell Metabolism

Paracellular transport requires nutrients and drugs to pass the enterocytes before becoming systemically available. Metabolism in IECs has gained increasing attention, not only due to the expression of key drug metabolizing enzymes (CYPs, UGTs) as mentioned before [2]. IEC and whole-body metabolism are tightly interrelated via production of incretin hormones by enteroendocrine cells [79] or factors like Fgf15 by enterocytes [80], respectively. Additionally, IECs are targets of remote-tissue metabolic signals such as insulin and leptin signaling [81,82]. In the gastrointestinal tract, carbohydrates, peptides and lipids are broken down with the help of digestive enzymes and absorbed by enterocytes. Subsequently, nutrients are either directly, or after interconversions, distributed in the body through the systemic circulation or serve as substrates for cellular metabolism or energy generation. Thus, IEC metabolism determines quality and availability of nutrients, constituting an initial check point before entering the whole organism. In this context, the intestinal microbiome has another key role, producing important metabolites such as short chain fatty acids (SCFAs), e.g., butyrate. Moreover, nutrient abundance and IEC metabolism is associated with pathologies and tissue homeostasis, for example high-fat diets were shown to enhance tumorigenicity of intestinal progenitors [83], and SCFAs as well as lactate promote intestinal healing processes [34,84]. Despite the fact that the general metabolic functions of enterocytes are known, many open questions remain on intestinal metabolic capacities and their relevance for health. It is not clarified whether the small intestine can act as a site for gluconeogenesis, which seems to be species-dependent [85,86,87] or how carbohydrate and lipid absorption and metabolism interact. Of note, sensing of dietary fat via fatty acid oxidation in enterocytes has been implicated in the control of eating [5], and modulation of enterocyte metabolism might affect whole-body glucose homeostasis and the development of diet-induced obesity [6,88]. Next to the lumen, IECs are also a site of presystemic drug metabolism and intestinal phase I metabolism has been recognized as a major determinant of oral drug bioavailability [89]. Clinical studies demonstrated that inhibition of CYP3A4-mediated intestinal metabolism can significantly improve oral bioavailability of a wide range of drugs [89], yet a common approach for characterization and quantitative prediction of intestinal metabolism is lacking, especially in the light that species differences profoundly impact drug metabolism within IECs [90]. Here, human organoids, especially from donors with different metabolic backgrounds are of great value, as they are amenable to mass-spectrometry-based methods such as lipidomics, allowing to map metabolism in a holistic manner.

## 5. Currently Used Models

A comprehensive in vitro model of the intestinal epithelium has been lacking for a long time, since neither cultivation of primary cells or tissues nor cell lines resembles the diversity and complexity of the intestine. Additionally, both primary culture and cell lines suffer from substantial drawbacks.

Primary intestinal cell cultures can be generated from human tissue samples derived from the small or large intestine [91]. Derived from isolated primary intestinal crypts, this type of culture provides a lot of advantages for investigating intestinal epithelial physiology [92]. Conserving the location-specific functions of their site of origin, such as distinct IEC-subtypes, primary crypt cultures represent a good model to study certain aspects of IEC function, e.g., intestinal nutrient sensing and molecular mechanisms underlying the secretion of gastrointestinal hormones. However, absorptive enterocytes contained in this type of culture remain in a poorly differentiated state, resulting in non-detectable levels of nutrient transporter expression and lack of nutrient absorption. Furthermore, primary crypt cells can only be kept in culture for a few days, maximum, as cells tend to dedifferentiate and degrade almost immediately. Hence, this system is mostly used as overnight culture and is not suitable for long-term culture or expansion [13], or for screening approaches, as availability of human tissue is restricted due to ethical and organizational reasons.

### 5.1. Cell Lines

The inherent problem of cell lines is their origin from tumorous tissue or the genetic modifications introduced to render them cultivable over long time periods. The downside to these alterations improving cultivability comprise a certain distance from the native tissue and genetic instability/susceptibility to mutations. Moreover, most cell lines are restricted to only one subtype of IECs. Nevertheless, immortalized cell lines are widely used for in vitro research on molecular and cellular processes including drug absorption, since primary IECs tend to go into apoptosis rapidly, therefore cultivation is only possible for a very limited time frame. The advantage of cell lines is that they are well-established, easy in handling and experiments are cost- and time-effective. As they are widely used in research, numerous protocols and experimental results are available in scientific literature. IEC lines that are commonly used in gastrointestinal research to evaluate permeability properties of drugs include the human Caco-2 and HT-29 cell lines as well as Madin–Darby canine kidney cell culture (MDCK). Both human cell lines are derived from colorectal adenocarcinomas [93], yet, as Caco-2 cells possess some features of small intestinal epithelial cells, they are commonly used as a model of the small intestine [94,95,96]. However, comparing drug transporter expression levels in normal colon and colon cancer to Caco-2, the expression pattern of Caco-2 cells was found to most closely resemble the gene expression profile within the normal colon, suggesting Caco-2 cells rather as an model of colonic drug adsorption [97]. Importantly, two major aspects determining oral bioavailability are not reflected by the Caco-2 model: this cell line exhibits tighter junctions compared to the small intestine of humans [98] and Caco-2 cells are not appropriate for evaluating active, carrier-mediated drug absorption due altered transporter expression patterns [99]. Similarly, many intestinal cell lines do not reflect the physiological expression of essential nutrient transporters like PEPT1 or SGLT1; thus, they do not exhibit the major characteristics of small intestinal enterocytes. In summary, due to their tumour-derived nature, functional as well as phenotypic characteristics differ highly from native human enterocytes [100], classifying them as a rather artificial model system. Another drawback of cell lines is the extremely high lab-to-lab variability of the experimental outcomes, in particular with Caco-2 cells [100,101]. Constant attempts have been undertaken to improve the Caco-2 model, for instance by integrating other cell types like fibroblasts [102] or mucin-secreting HT29-MTX cells [103]. However, neither conserving essential epithelial properties nor region-specific functions, IEC lines offer only limited possibilities to study the complex epithelial cell biology and processes involved in oral bioavailability, especially with regard to the complex interdependencies of transporters for efficient functioning.

### 5.2. Other Models

Beyond the IEC layer, models with increased complexity as well as reduced models exist for evaluating drug absorption in a pre-clinical setup. An example for a simplified model is the parallel artificial membrane permeability assay (PAMPA), a high-throughput screening (HTS) technique developed to predict passive permeability through numerous different biological membranes, such as the gastrointestinal tract, blood brain barrier, or the dermal layer. Based on an artificial membrane separating two compartments different environmental factors can be tested for their impact on absorption and distribution kinetics of compounds [104]. PAMPAs are a valuable tool to investigate physical aspects of pharmacokinetics, yet these models cannot be applied to complex scientific questions involving cellular functions and metabolism. More complex models comprise rodent explant models like the side-by-side diffusion chamber (Ussing chamber) and everted gut sac models, as well as in vivo animal studies. Whole-tissue explants display properties such as expression and activity of nutrient transporters similar to the small intestine of rodents [105]. However, tissue explants have a poor stability and very limited viability in vitro (max. a few hours), hence the use of this system is clearly restricted to short-term experiments. In addition, primary tissue explants such as everted gut rings contain different cell types such as muscle, connective tissue, immune cells and neurons next to IECs. Depending on the scientific question, this might be a limitation, for example if specific processes in IECs are in the focus of research. Ussing chamber approaches mainly use excised rat or other rodent tissue, hence better reflect physiology than cell lines but are limited in transferability due to species differences. Consequently, results from Caco-2 monolayers and Ussing chamber experiments are poorly correlative [72,99,106]. Monkeys, dogs, rats and mice are frequently used for in vivo studies on drug bioavailability and intestinal absorption studies. However, as mentioned before, expression patterns of drug metabolizing enzymes and drug transporters as well as intestinal physiology differ strongly from human biology [107,108], and these pronounced interspecies differences result in poor transferability of experimental data to humans [109,110]. Common to these models is the large number of animals needed for screening, causing high costs and raising ethical questions regarding the limited output/use of the experiments.

**Figure 2:**
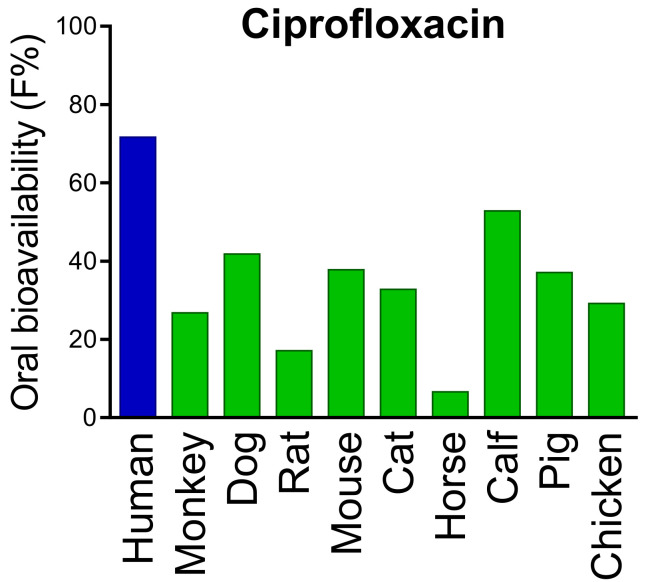
Inter-species differences in oral bioavailability of ciprofloxacin. Data are obtained from the PACT-F database which contains drug structures and detailed pharmacokinetic results from more than 25,000 trials. Pronounced inter-species differences are also found for many “blockbuster drugs” Adapted from [110], Blockbuster Drugs 2021.

For widespread diseases such as diabetes or cancer, drug development success rates are particularly low [111,112]. In general, only 5 to 10 percent of drugs make it to the market, despite being proven as safe and effective in preclinical animal studies [113,114] (Table 1). The high failure rate is attributed to species-specific differences and hence poor transferability from animal models to humans [115,116,117] (Figure 2). In fact, machine learning and computational models have already been shown to predict human oral bioavailability more precisely than animal trials [118,119,120]. Furthermore, commercially and freely available expert systems such as IMPACT-F [121] or SwissADME [122] combine large and comprehensively annotated knowledge databases containing drug structures and detailed pharmacokinetics with artificial intelligence and machine learning to predict pharmacokinetics of novel drug candidates solely from compound structure [118]. Hence, oral bioavailability and plasma protein binding of drug candidates can be evaluated in silico before human clinical trials are carried out. These expert systems are already in use by pharmaceutical companies in different therapeutic areas such as diabetes, inflammation, antivirals, autoimmune diseases and cancer for lead-optimization and prioritisation of drug candidates and to calculate first-in-human dose (FiH) in clinical trials [123]. Thus, new in silico methods foster present and future drug discovery and development, improving efficiency and safety of human clinical trials [124].

## 6. Human Intestinal Organoids and How They Can Improve Drug Screening

In 2009, Sato et al. reported on the generation and long-term ex vivo cultivation of intestinal organoids [130]. This method represented a breakthrough for biomedical research including stem cell biology, developmental biology, basic medical science, disease modelling and personalized medicine. The possibility of long-term culture and expansion is particularly critical for scientific questions and applications including screening approaches, as human tissue availability is restricted owing to organizational and ethical reasons [131]. Due to the enormous potential of organoids as preclinical models, for personalized medicine as well as high-throughput drug screening [132], organoid biobanks have been established collecting diseased organoids and matched normal organoids from large numbers of individuals [133,134]. It is essential for pharmaceutical applications that the human disease is reflected in vitro/ex vivo as close as possible. Organoids foster the establishment of improved, “real” disease models for experimental research [135]. Cerebral organoids, also known as mini brains, can be generated from patients suffering from Alzheimer’s or Parkinson’s disease [136,137], pancreatic organoids from diabetes patients [138], intestinal organoids from people suffering from inflammatory bowel diseases [139], and 3D lung models from smokers, COPD (chronic obstructive pulmonary disease) or asthma patients [140]. These models offer numerous applications and have boosted biomedical research in recent years. Organoid biobanks have already facilitated tumour drug screening approaches and organoids are optimal preclinical models for pharmacodynamic profiling of human tumours [141] and quick selection and validation of drugs in personalized medicine [142,143]. In fact, in 2015 the first patients received personalized treatment for cystic fibrosis solely on the basis of screening drugs using intestinal organoids [144,145]. To date, organoid biobanks have been obtained from various tumour tissues, including gastric [146], colonic [133], liver [147] and kidney cancer [148]. Some of these models such as human airway, ocular, intestine or skin models are already commercially available and even accepted for regulatory testing. Other complex organoid models such as the cystic fibrosis models are not yet commercially used but in a preclinical state and for basic or applied research. In contrast, personalized cancer treatment based on microtumours generated from patients in order to define the most effective therapy are commercialized and already applied in the clinical field [149]. However, ethical standards remain to be defined for commercial use of organoid biobanks [134].

### 6.1. Properties of Intestinal Organoids

**Figure 3:**
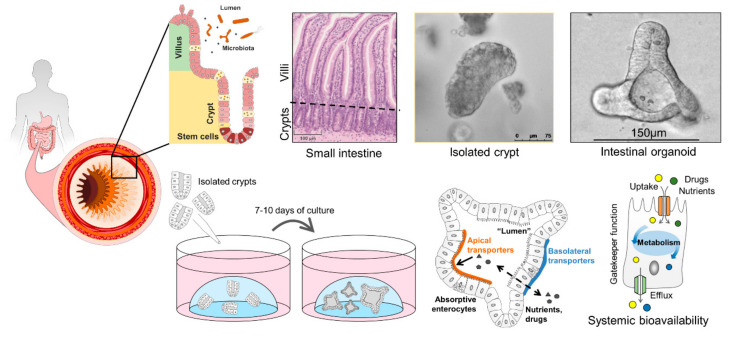
Human intestinal organoids can be generated from isolated primary crypts and constitute a superior model to study intestinal transport processes and intestinal epithelial cell metabolism as gatekeeper for systemic bioavailability of nutrients and drugs.

Thus, organoids represent a superior model of the intestinal epithelium, and can be additionally used for basic studies on nutrient and drug absorption, oral bioavailability and toxicology testing [150,151]. To generate human intestinal organoids, induced pluripotent stem cells (iPSCs) [152] and isolated crypts from biopsies or surgical specimen can be used [153] (Figure 3). Depending on the experimental method that is used to grow intestinal organoids, they develop slightly different properties in culture and are sometimes referred to as organoids (if grown from stem cells) or enteroids or adult intestinal organoids (if grown from crypts) [154]. A nomenclature has been proposed to distinguish between different ex vivo intestinal cultures [155], however the vast majority of scientific publications simply uses the term organoids to describe mini guts (or mini organs) grown in vitro. Intestinal organoids are amenable to all standard molecular biological techniques for manipulation such as controlled gene expression [156], and genetic engineering approaches like the CRISPR/Cas9 technology [157]. Regarding downstream readouts, they are compatible with virtually all methods used to analyse cells and/or tissue samples [158] including different approaches for gene- and protein expression analyses [8], live cell imaging [10] and metabolic measurements including direct respirometry and mass spectrometry-based methods [159]. Using certain modulators like the γ-secretase inhibitor or RANKL, organoid differentiation can be steered into generation of distinct intestinal epithelial cell (IEC) subtypes like EECs [160] or microfold (M) cells [161]. Alternatively, withdrawal of compounds like Wnt3a or R-Spondin1 leads to general differentiation processes including maturation of the enterocyte linage [75,162], boosting expression of nutrient transporters and associated proteins [9].

### 6.2. Intestinal Organoids to Assess Nutrient and Drug Uptake

**Figure 4:**
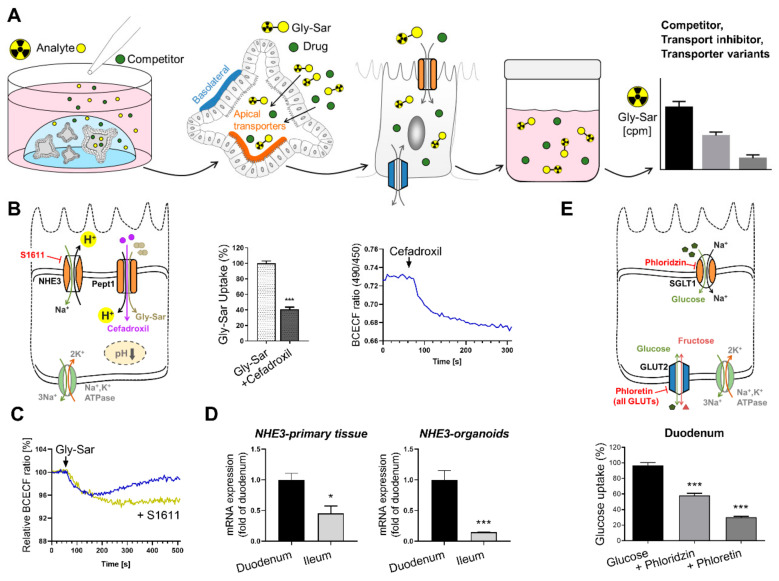
Workflow to study intestinal transport processes and downstream events in human intestinal organoids. (**A**) Schematic representation of the experimental procedures for measuring transport activity using radiolabeled substrates. (**B**) Left: schematic illustration of the transporters investigated, and inhibitor used. Middle: reduction of radiolabeled Gly-Sar (model substrate for PEPT1) uptake using the antibiotic cefadroxil as competitive inhibitor. Right: course of intracellular acidification induced by cefadroxil exposure. (**C**) Course of intracellular acidification induced by Gly-Sar exposure with the NHE3-inhibitor S1611, preventing reestablishment of cellular pH. (**D**) mRNA expression of NHE3 in primary tissue and organoids derived from different intestinal segments demonstrating region-specific gene expression in organoids. (**E**) Upper panel: Schematic illustration of the transporters investigated, and inhibitors used. Lower panel: uptake of radiolabeled glucose in organoids derived from duodenal tissue. Asterisks indicate significant differences * *p* < 0.05, *** *p* < 0.001; Figures and graphs adapted from [9], Front. Bioeng. Biotechnol. 2020.

Recently, we demonstrated the suitability of human intestinal organoids for studying nutrient and drug uptake, visualization of transport processes and metabolic analyses [9] (Figure 4). Here, we validated the transporter-mediated uptake of small cyclic hexapeptides used as anti-cancer drugs and of the β-lactam antibiotic cefadroxil. Although still at the stage of basic/applied research, these data highlight intestinal organoids as a pharmacologically relevant screening tool for transporter-mediated uptake of drug candidates. This aspect of oral bioavailability might have been underappreciated previously in Caco-2 assays, due to lack of physiological transporter expression. Furthermore, intestinal organoids offer the possibility to evaluate efflux processes that limit drug absorption [44] and also the distinct role of the ion pump NHE3 in Na^+^ absorption has been explored in human enteroids under normal and diseased conditions (diarrhoea) [75]. To elucidate intestinal transport processes on a functional level, not only transport of compounds but also downstream effects on intracellular signalling need to be monitored. As mentioned before, peptide transport over the plasma membrane occurs in cotransport with protons, leading to cytosolic acidification of enterocytes, a process that can be visualized by live-cell imaging using fluorescent probes [10,74] (Figure 4). Also, many transporter activities (e.g., PEPT1) [163], intracellular translocation events (e.g., GLUT2) [164] and receptor activation involve intracellular calcium signalling that can be visualized in the same way using the calcium-indicator Fura-2-AM. Hence, intracellular changes immediately reflect transport activities, providing direct evidence for substrate fluxes or for activation of receptors by a putative ligand/drug. Therefore, live-cell imaging of intracellular events is routinely applied in pharmacological screenings in simple cell cultures and our results on the visualization of transport and signalling processes [9] emphasize the high-resolution measurements possible in intestinal organoids.

Importantly, these readouts and metabolic measurements can be performed in intestinal organoids [9], providing a holistic tool to study drug uptake and epithelial metabolism of drugs in parallel. In summary, human intestinal organoids come closer to physiology than any other cell-based model and are superior to animal (rodent)-derived organoids and (cancer) cell lines. Especially in the context of metabolism and diseases, human organoids are the tool of choice, since metabolic properties profoundly differ between species and alterations in cellular metabolism are associated with many pathologies. Thus, human organoids hold great potential to answer remaining questions on intestinal metabolism and to identify drug targets to improve overall metabolic health.

### 6.3. Current and Future Strategies for Organoids in Pharmacological Research

Already simple organoid culture protocols hold great potential for improving the toolbox of pharmacologic and biomedical research. The most basic and probably least cost- and labour extensive culture protocol, organoids embedded in a laminin-gel matrix and cultured in 3D [9], is readily compatible with methods to assess uptake and monitor cellular consequences [9,10]. Beyond these applications, various other readouts are possible in this setup, for example assessment of proteasome activity [9], which is of interest in the context of proteasome inhibitors, an important class of drugs in the treatment of cancer [165]. Additionally, adapting culture protocols to certain applications, for example changing the medium composition to promote cell differentiation or to “break up” organoids prior to uptake studies offer possibilities to improve results in parallel to reducing costs. Particularly for transport studies, the fact that all substances added to the culture medium first reach the “outside” of the organoid, the basolateral side, is a major drawback, as well as that a directed transport cannot be investigated in 3D organoids. A possible solution to overcome these hurdles are intestinal organoids with reversed polarity (in which the apical side faces outwards) due to the lack of extracellular matrix [166] or organoid cells seeded on Transwell inserts to form a 2D cell lawn that is accessible from both the basolateral and apical side [8,167]. Once more highlighting the importance of organoid-based systems for the assessment of oral bioavailability, paracellular transport of fluorescein, transcellular transport of propranolol, and basolateral efflux of rhodamin123, a substrate of P-glycoprotein, has been measured in a proof-of-concept study in a system based on human organoid-derived cells seeded on a physiological scaffold to form a 2D monolayer [168]. The field of applications for organoids is still rapidly growing, and there is a trend towards more complex and sophisticated organoid-based model systems. On the one side, there are approaches to implement different cell types like neurons, immune cells and mesenchymal cells in organoid culture systems, on the other side, co-cultures with bacterial and viral pathogens and commensals are established [169,170]. In general, there is a trend towards reproducing the complex tissue environment, as the importance of oxygenation, mechanical stress, and tissue communication via soluble factors for tissue maturation and maintenance is more and more appreciated [41].

Human organoids combined with the organ-on-a-chip technology offer a robust and reliable system to study human-relevant processes [171]. The organ-on-a-chip (OOC) technology holds great potential particularly for drug development and toxicological screenings using organoids or other three-dimensional cell models derived from multiple tissues [169]. OOC systems, also known as microphysiological systems (MPS) or multi organ chips (MOC), are biochip devices containing up to ten organ-like cell models that interact via a microperfusion system mimicking the blood and urinary circuits [172]. The biochip contains a micro pump, which regulates the flow rate and optional elements for the adjustment of physiological parameters such as pH or oxygen supply. Organ models display an active metabolism and are used for testing systemic effects of drugs or chemicals delivered to the microfluidic system. The effects of the applied substance of interest can be investigated in blood or urinary samples taken from multiple parts of the microfluidic system and additionally from the tissue models by removing them from the chip for further experimental analyses. Pharmacokinetics as well as bioavailability, metabolism and excretion of drugs or toxic compounds can hence be studied in detail in a human-relevant manner [173,174].

## 7. Regulatory Aspects

MPS offer numerous advantages highly appreciated by the pharmaceutical industry including reliability of the experimental outcomes, time and cost effectiveness and high throughput scaling [175]. Furthermore, MPS-based testing of drug candidates requires lower quantities of test substance compared to in vivo animal studies. This is an economic advantage for pharmaceutical companies as the production of novel pharmacological compounds is associated with high costs. Therefore, a number of global pharmaceutical companies are running R&D programs in collaboration with research institutes and MPS suppliers to develop and optimize advanced organoid-based models and MPS approaches.

Some countries have already developed concrete strategic roadmaps to phase out animal experiments for regulatory purposes. In the Netherlands, animal experiments are supposed to be banned in the regulatory safety assessment of chemicals, drugs, food additives etc. by 2025 [176]. Also, the UK and Norway have developed strategic papers for replacing animal studies by NAMs [177,178]. The FDA (U.S. Food and Drug Administration) has developed a toxicological roadmap for the replacement of animal testing by alternative methods in the regulatory sector [179] and is running several research programs on animal-free systems such as MPS. In collaboration with the MPS developers, the FDA is evaluating chip systems for use on pharmaceuticals and other products that are reviewed and approved by regulatory authorities to improve human health. Test systems includes lung chips for investigating protective immunity and vaccine safety, in particular related to COVID-19, liver chips to predict patient susceptibility and adaptation to drug-induced liver toxicity and brain chips to study Alzheimer’s disease [180,181]. All systems are using human-/patient-derived iPSCs.

Human safety assessment is crucial in the regulatory sector when it comes to toxicological evaluation of novel food compounds, medical products and other consumer products. Yet, existing in vivo animal models, e.g., to test skin irritation, suffer from high variability and poor reproducibility [182,183]. Consistently, new approach methods (NAMs) for toxicity tests, technologies and approaches that can potentially provide the same hazard and risk assessment information without the use of animal testing, are currently developed [184,185,186]. An overview of non-animal methods from different scientific areas is provided by the NAT database (www.nat-database.org).

This is of particular interest, as animal experiments have been banned for the testing of cosmetics in the European Union and marketing of cosmetic products tested on animals is prohibited since 2014. Likewise, the Food and Drug Administration (FDA) of the United States promotes validation of human organoid models and MPS for regulatory purposes in collaboration with MPS suppliers [187]. Although the vision of animal-free research is close, a lot of work is left to establish new standards and to fully replace animal-based models in the regulatory sector. Despite the fact that animal models often poorly predict human physiology, they are established and currently still serve as the gold standard in a number of regulatory guidelines. Interdisciplinary communities of stakeholders representing research institutes, pharma and other companies, regulators, politicians and NGOs have been constantly working on strategies to increasingly and finally fully replace animal models within regulatory guidelines. In the EU, the validation of non-animal methods is coordinated by the European Centre for the Validation of Alternative Methods (ECVAM) which belongs to the Joint Research Centre (JRC) and consequently to the European Commission. ECVAM is responsible for the assessment of in vitro methods for regulatory risk assessment and works in close collaboration with the OECD and with several scientific expert committees and stakeholder groups. A series of scientific reviews have been published by the European Commission via ECVAM describing thousands of in vitro models for different medical fields such as respiratory diseases [188] or breast cancer [189]. ECVAM is continuously evaluating new in vitro systems for the replacement of in vivo animal testing as can be viewed in the TSAR database (www.tsar.de), such as advanced tissue cultures of the human skin and the respiratory tract or ocular models. These 3D cell models are accepted for safety assessment many of them are commercially available. Grown from primary human cells, such cultures closely reflect the properties of native human epithelia. Skin models representing different ethnic groups with different amounts of melanocytes are available as well as lung models derived from different regions of the human airway system such as bronchial or alveolar models. These models can be constructed not only by hand but also by 3D bioprinting technologies enabling high accuracy and reproducible conditions [190,191,192]. Additionally, bioprinting enables generation of artificial human skin equivalents with complex structures including vascularisation, hair follicles and sweat glands. Although a number of NAMs have already been accepted for regulatory purposes at the EU level, the validation processes for these methods take too long (many years) and needs to be accelerated.

## 8. Future Directions

MPS and organoid models can be combined with in silico approaches, complex computer-based models that precisely predict ADME processes and pharmacokinetics in pharmaceutical and toxicological research [193]. It has been proven that in silico approaches even provide a better predictability in toxicology testing compared to animal experiments [194,195]. Computational methods can also be used as alternatives to animal testing in safety and efficacy testing such as Quantitative Structure–Activity Relationship (QSAR) modelling and physiologically based kinetic and dynamic modelling. QSAR models predict biological and toxicological effects of drugs and other chemicals based on their physicochemical and structural properties. Chemical properties can also be predicted by “read-across”, grouping of chemicals on the basis of structural and biological similarity. Read-across is typically carried out in addition to QSAR, to increase the overall confidence in the predicted properties. Complementary, PBK models predict the distribution of a drug or chemical in a living organism. PKB models are used to interpret in vitro toxicity data simulating internal concentrations following exposure to the chemical via the diet, skin or inhalation. Coupling PBK with other mathematical models describing biological responses in a specific organ is called physiologically based kinetic and dynamic (PBKD) modelling. The virtual cell-based assay (VCBA) is a mathematical model developed by the JRC [196]. It simulates the distribution and biological effects of chemicals in a range of in vitro systems. Integrated approaches combining computational modelling, human studies and human-based in vitro models, such as advanced cell cultures or MPS will improve human safety assessment and accelerate medical development.

## 9. Conclusions

There is no doubt that human-based model systems are needed to produce human-relevant data for medical, pharmacological or toxicological purposes. High failure rates within the drug development pipeline constitute a problem increasingly recognised and targeted by the scientific community including academia, industry and regulatory bodies [197,198,199,200,201]. Failure rates calculated based on statistical analyses of drug approvals in a certain time frame account to as much as 95% [117,127,129], with oncology, neurology and cardiovascular diseases displaying the worst results [129]. To a large extent, this failure which occurs mainly in safety and efficacy testing, can be attributed to a lack of transferability of preclinical data including animal experiments to the human trials. Computational approaches together with advanced in vitro models complemented by human clinical and epidemiological studies offer a research portfolio that is capable of reliably investigating human-relevant issues related to health and disease. We are on a good way having human-based techniques available that provide excellent tools to study drug bioavailability and other pharmaceutical issues in a human-relevant manner.

## Figures and Tables

**Table 1:** Failure rates of substances entering Phase I trials.

Failure Rate	No of Drugs Tested	Years	Reference
81–84%	1.738	1993–2004	DiMasi et al., 2010 [125]
88.6%	>9.200	1996–2014	Smietana et al., 2016 [126]
86.2%	21.143	2000–2015	Wong et al., 2019 [127]
90–95%	>850	2002–2008	Arrowsmith, 2012 [113]
89.6%	>7.300	2003–2011	Hay et al., 2014 [128]
90.4%	7.455	2006–2015	Thomas et al., 2016 [129]

## Data Availability

Data sharing not applicable.

## Abbreviations

ADME	(Drug) Absorption, Distribution, Metabolism, and Excretion
ECVAM	European Centre for the Validation of Alternative Methods
FDA	U.S. Food and Drug Administration
HTS	High-Throughput Screening
IECs	Intestinal Epithelial Cells
JRC	Joint Research Centre
MOC	Multi-Organ Chip
MPS	MicroPhysiological System
NAMs	New Approach Methods
NAT	Non-Animal Methods
OOC	Organ-On-a-Chip
PAMPA	Parallel Artificial Membrane Permeability Assay
PBKD	Physiologically Based Kinetic and Dynamic
QSAR	Quantitative Structure-Activity Relationship
VCBA	Virtual Cell Based Assay
